# Identification and characterization of antigen-specific CD4^+^ T cells targeting renally expressed antigens in human lupus nephritis with two independent methods

**DOI:** 10.1038/s41598-020-78223-3

**Published:** 2020-12-04

**Authors:** Sebastian Tesch, Dimas Abdirama, Anna-Sophie Grießbach, Hannah Antonia Brand, Nina Goerlich, Jens Y. Humrich, Petra Bacher, Falk Hiepe, Gabriela Riemekasten, Philipp Enghard

**Affiliations:** 1 Department of Nephrology and Medical Intensive Care, Charité—Universitätsmedizin Berlin, Corporate member of Freie Universität Berlin, Humboldt-Universität Zu Berlin, Berlin Institute of Health, Berlin, Germany; 2 Deutsches Rheuma-Forschungszentrum, A Leibniz Institute, Berlin, Germany; 3 Department of Rheumatology and Clinical Immunology, Charité—Universitätsmedizin Berlin, Corporate member of Freie Universität Berlin, Humboldt-Universität Zu Berlin, Berlin Institute of Health, Berlin, Germany; 4 Department of Rheumatology and Clinical Immunology, Universitätsklinikum Schleswig-Holstein, Lübeck, Germany; 5 Institute of Immunology, Christian-Albrechts Universität zu Kiel and Universitätsklinik Schleswig-Holstein, Kiel, Germany; 6 Institute of Clinical Molecular Biology, Christian-Albrechts Universität zu Kiel, Kiel, Germany

**Keywords:** Immunology, Diseases, Medical research, Nephrology, Rheumatology

## Abstract

In the search for anti-renal autoreactivity in human lupus nephritis, we stimulated blood-derived CD4^+^ T cells from patients with systemic lupus erythematosus with various kidney lysates. Although only minor responses were detectable, these experiments led to the development of a search algorithm that combined autoantibody association with human lupus nephritis and target gene expression in inflamed kidneys. Applying this algorithm, five potential T cell antigens were identified. Blood-derived CD4^+^ T cells were then stimulated with these antigens. The cells were magnetically enriched prior to measurement with flow cytometry to facilitate the detection of very rare autoantigen-specific cells. The detected responses were dominated by IFN-γ-producing CD4^+^ T cells. Additionally, IL-10-producing CD4^+^ T cells were found. In a next step, T cell reactivity to each single antigen was independently evaluated with T cell libraries and [^3^H]-thymidine incorporation assays. Here, Vimentin and Annexin A2 were identified as the main T cell targets. Finally, Vimentin reactive T cells were also found in the urine of three patients with active disease. Overall, our experiments show that antigen-specific CD4^+^ T cells targeting renally expressed antigens arise in human lupus nephritis and correlate with disease activity and are mainly of the Th1 subset.

## Introduction

Lupus nephritis (LN) is one of the most serious complications of systemic lupus erythematosus (SLE)^1,2^. Affecting approximately 50% of SLE patients, it is a major contributor to disease-associated morbidity and mortality^3,4^.

In LN immune complexes, complement and antibodies are deposited in the glomeruli of the kidneys, leading to local inflammation. Thus, LN has been recognized as a prototypical glomerulonephritis, with diagnosis and therapy focusing mainly on glomerular involvement^5–7^. However, in addition to glomerular damage, T cell-rich infiltration in the kidney interstitium takes place^8,9^. A major part of this infiltrate consists of CD4^+^ T cells, and the amount of infiltration has been shown to correlate with kidney damage^10,11^. In addition, reports of local MHC class II upregulation on epithelial cells in the kidneys of LN patients and an intrarenal oligoclonal T cell repertoire both suggest the involvement of antigen-specific T cells^12,13^.

Various mouse studies further strengthen this concept. In one study, B cells of lupus-prone MRL.lpr mice were genetically modified so that they were unable to produce antibodies. Interestingly, these mice still developed LN with a T cell-rich infiltration driving inflammation in the absence of autoantibody production^14^. Another study reported the induction of interstitial lesions in recipient mice solely by T cell transfer^15^. Additionally, IFN-γ-producing CD4^+^ T cell clones that were isolated from the kidneys of lupus-prone mice were sufficient to elicit nephritis in recipients upon cell transfer^16^.

Despite these observations suggestive of an anti-renal CD4^+^ T cell involvement in LN, at present, little is known about their antigen specificity. Here, we report the identification of an antigen-specific CD4^+^ T cell response from patients with active LN against five potential renal antigens utilizing flow cytometry as well as T cell libraries.

## Results

### ***Only marginal reactivity of CD4***^+^***T cells from SLE patients against kidney lysates***

To detect an anti-renal T cell response, we stimulated CD4^+^ T cells from SLE patients with various lysates from healthy kidney tissues. The upregulation of activation markers (CD69 and CD154) and the expression of IFN-γ were assessed to detect antigen-specific T cells.

**Figure 1:**
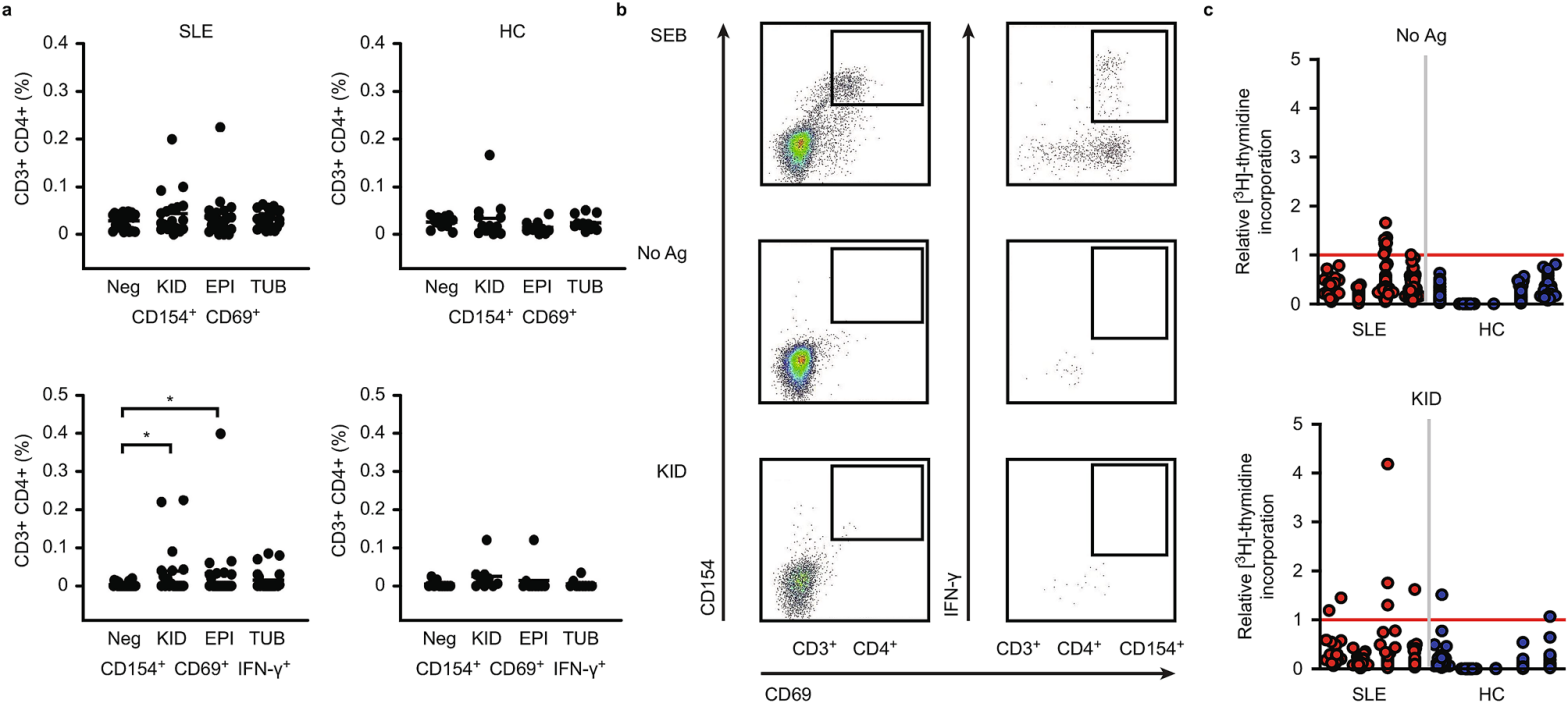
Only marginal reactivity of CD4^+^ T cells from SLE patients against different kidney lysates. (**a**) Respective frequencies of CD3^+^CD4^+^ T cells expressing activation markers CD154, CD69 and IFN-γ after 6 h PBMC stimulation with three different kidney lysates (KID, EPI, TUB) and measurement with flow cytometry (n = 23 SLE, n = 12 HCs). (**b**) Dot plot from one patient with acute LN after stimulation with SEB (positive control), no Ag and KID (**c**)**.** CD4^+^ T cell libraries from 4 SLE patients and 5 HCs stimulated with no Ag or KID and measurement of [^3^H]-thymidine incorporation. Each column shows the relative [^3^H]-thymidine incorporation from a single patient, and each dot represents one microculture in a 96-well plate. Microcultures with a relative [^3^H]-thymidine incorporation above one (red line) were assumed to contain one reactive T cell. Libraries were set up with 2000 T cells per well. The Wilcoxon matched-pairs signed ranked test was used for statistical analysis (*p < 0.05), **p < 0.01, ***p < 0.001). Horizontal lines depict the median. *SLE* systemic lupus erythematosus, *HC* healthy control, *PBMC* peripheral blood mononuclear cells, *KID* human kidney normal tissue lysate, *EPI* human renal tubular epithelial cell lysate, *TUB* human renal proximal tubular epithelial cell lysate, *SEB* staphylococcal enterotoxin B.

No differences in the number of CD154/CD69-positive T cells were observed upon stimulation with all kidney lysates (Fig. 1a). However, after stimulation with total kidney lysate and tubular epithelial cell lysate, a marginal increase in the number of CD154/CD69/IFN-γ triple-positive T cells was found (n = 23, Wilcoxon matched pairs test, p = 0.01 and p = 0.04, respectively, Fig. 1a). These differences were not detected in healthy controls (HCs, n = 12). The T cell superantigen staphylococcal enterotoxin B (SEB), which was used as a positive control, provoked significant differences from unstimulated samples in all tested individuals (exemplary dot plot in Fig. 1b; frequencies for SLE patients shown in Supplemental Fig. 1h)^17^.

To further evaluate the anti-renal reactivity, CD4^+^ T cells from four SLE patients and five HCs were expanded and stimulated with the more promising lysate KID in T cell libraries. Again, only marginal reactivity against KID was detectable (Fig. 1c). The applied analysis algorithm showed no significant differences in terms of autoreactive T cell numbers between SLE patients and HCs (Supplemental Fig. 1h). However, the relative [^3^H]-thymidine incorporation data shown in Fig. 1c suggest slightly higher reactivity against KID in SLE patients than HCs.

Consequently, we were not able to detect a convincing anti-renal T cell response using kidney lysates. However, the results were still indicative of such a response, possibly below the detection levels of the chosen approaches. Thus, to detect more subtle events, we increased accuracy in a next step by identifying target kidney antigens and applying antigen reactive T cell enrichment via CD154 beads.

### Identification of five potential target antigens

At present, no major T cell targets are known in LN. To identify potential renal T cell antigens, we used two assumptions. First, as SLE is characterized by the production of autoantibodies, we hypothesized that an anti-renal CD4^+^ T cell response would likely be accompanied by antibodies targeting the same antigens. Second, as lysates from healthy kidneys only elicited minor/no T cell responses, we assumed that the relevant T cell antigens are only upregulated upon kidney inflammation, as has been described for autoreactive B cells^18^. Applying both assumptions, five target antigens with increased expression and a strong association of the coherent autoantibody with LN development were identified: Vimentin, Annexin A1, Annexin A2, Ribosomal Protein P1, and Ribosomal Protein P2. A flowchart depicting the search algorithm is shown in Supplemental Fig. 1a.

### ***Antigen-specific CD4***^+^***T cells arise in LN and are mainly of the Th1 phenotype***

Next, we used flow cytometry to characterize the autoreactive T cell response against an antigen pool (Ag–P) containing all five identified antigens. Prior to measurement, cells were magnetically enriched for CD154, which is expressed by CD4^+^ T cells upon interaction with antigen-presenting cells. Thus, CD154 represents a marker for antigen-specific activation. This method has been termed antigen reactive T cell enrichment (ARTE) and enables the detection of low frequency antigen-specific T cells^19^.

**Figure 2:**
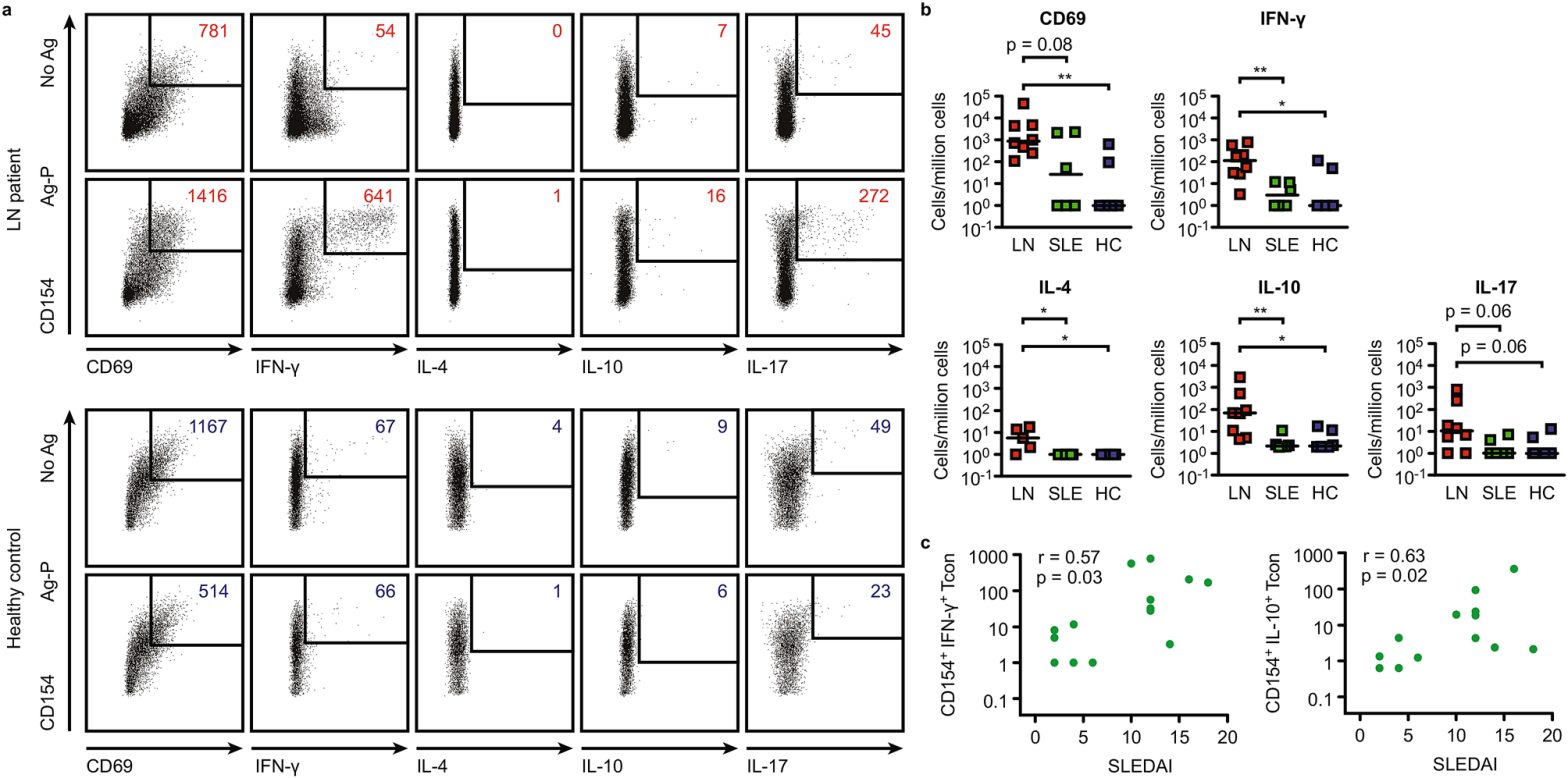
Renal autoantigen reactive CD4^+^ T cells are expanded in active LN patients. (**a**) Depiction of an example dot plot of CD3^+^CD4^+^ T cells from an active LN patient (top) and an HC (bottom). Cells were stimulated with Ag–P and subsequently enriched for CD154 via magnetic beads (ARTE method). Numbers indicate the cell count. (**b**) Calculated frequencies of CD69^+^, IFN-γ^+^, IL-4^+^, IL-10^+^, IL-17^+^ autoreactive CD154^+^ Tcons for active LN patients (n = 8), inactive SLE patients (n = 6) and HCs (n = 6). Horizontal lines depict the median. (**c**) Correlation of CD154^+^IFN-γ^+^ and CD154^+^IL-10^+^ Tcons with disease activity (SLEDAI, n = 14). The Mann–Whitney test was used for statistical analysis in (**b**), Spearman´s rank correlation in c. (*p < 0.05, **p < 0.01; r = Spearman´s rank correlation coefficient). Ag–P contained VIM, ANXA1, ANXA2, RPLP1 and RPLP2. *ARTE* antigen reactive T cell enrichment, *No Ag* no antigen, *Ag–P* antigen pool, *LN* lupus nephritis, *SLE* systemic lupus erythematosus, *HC* healthy control, *SLEDAI* systemic lupus disease activity index, *PBMC* peripheral blood mononuclear cells, *Tcon* conventional CD3- and CD4-positive T cells, *VIM* Vimentin, *ANXA1* Annexin A1, *ANXA2* Annexin A2, *RPLP1* Ribosomal Protein P1, *RPLP2* Ribosomal Protein P2.

In addition to CD154 and CD69, lineage determining cytokines for Th1 (IFN-γ), Th2 (IL-4) and Th17 (IL-17) T cells as well as IL-10 were measured^20^. An exemplary dot plot for one active LN patient and one HC is shown in Fig. 2a. Background cell numbers were subtracted from Ag–P stimulated samples, resulting frequencies are depicted in Fig. 2b, and cell frequencies without subtraction are shown in Supplemental Fig. 1i (n = 8 LN, 6 SLE and HCs).

More conventional CD3- and CD4-positive T cells (Tcons) expressing CD154 and CD69 were detected in active LN patients than in HCs (Mann–Whitney test p = 0.008). Differences between active and inactive patients were numerically different but did not reach statistical significance (Mann–Whitney test p = 0.08). Significantly more CD154^+^IFN-γ^+^ Tcons were found in LN patients than in inactive SLE patients or HCs (Mann–Whitney test p = 0.008 and p = 0.03, respectively), with a median cell number of 112 cells per million cells in LN patients. The same was true for CD154^+^IL-10^+^ Tcons (Mann–Whitney test p = 0.008 and p = 0.02, respectively) with a median cell number of 70 cells per million cells. Very few CD154^+^IL-4^+^ Tcons were detected in all three groups. Although differences between active LN patients and inactive SLE patients or HCs reached statistical significance (Mann–Whitney test for both p = 0.02), the detection was rather questionable due to extremely low overall cell frequencies. Differences in CD154^+^IL-17^+^ Tcon frequencies were almost statistically significant (Mann–Whitney test for both p = 0.07) with a median cell frequency of 10 cells per million cells. The cell frequencies of CD154^+^IFN-γ^+^ and CD154^+^IL-10^+^ autoreactive Tcons positively correlated with disease activity (systemic lupus erythematosus disease activity index, SLEDAI, n = 14), as determined by Spearman’s rank correlation coefficients (r = 0.57, p = 0.03 and r = 0.63, p = 0.02, respectively, Fig. 2c).

### Vimentin and Annexin A2 are the prominent antigens for active LN patients compared to inactive patients

**Figure 3:**
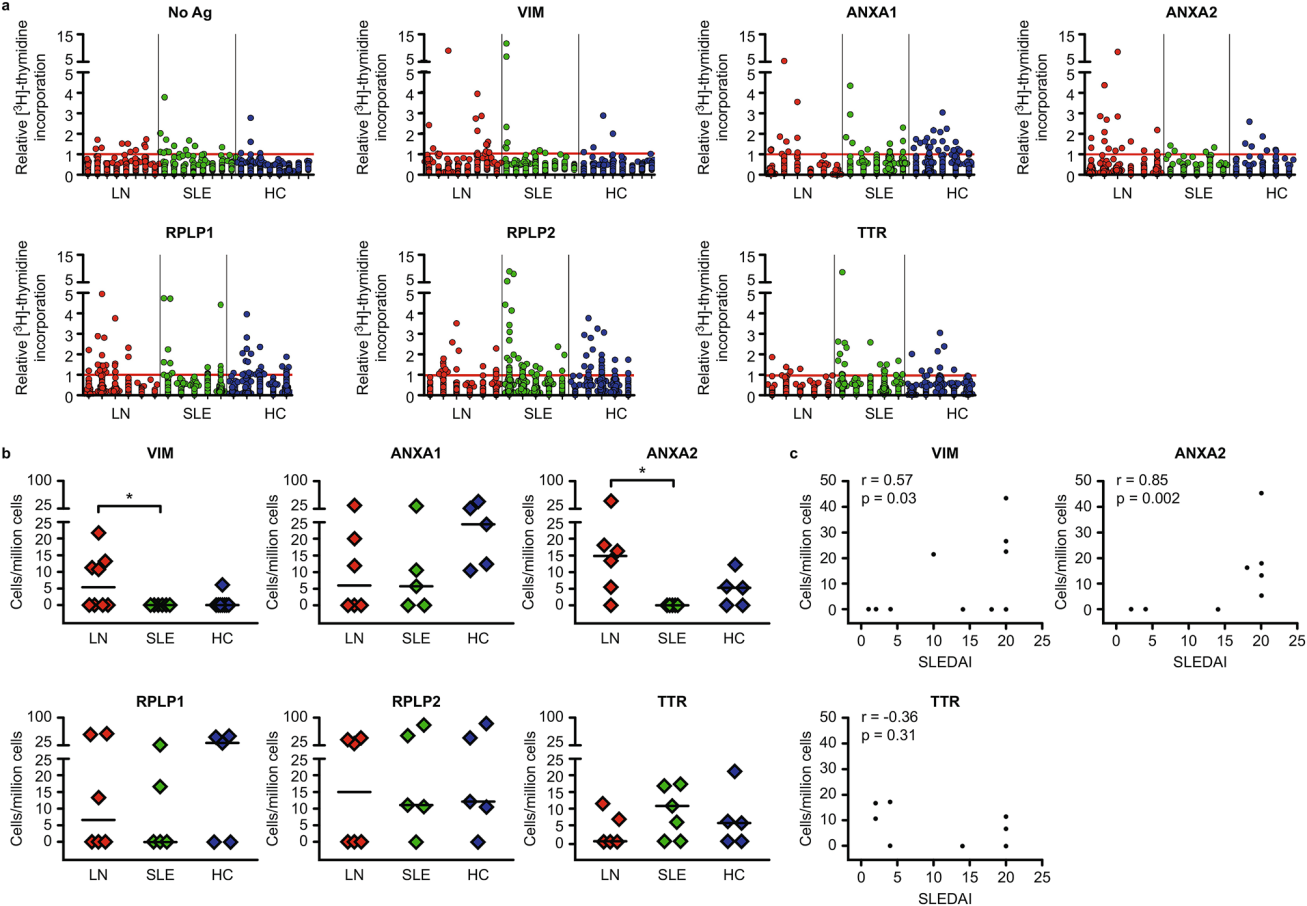
CD4^+^ T cell responses against single antigens. (**a**) CD4^+^ T cell libraries from active LN patients, inactive SLE patients and HCs were stimulated with single antigens. Each column shows the relative [^3^H]-thymidine incorporation from a single patient, and each dot represents one microculture in a 96-well plate. Microcultures with a relative [^3^H]-thymidine incorporation above one (red line) were assumed to contain one reactive T cell. Libraries were set up with 2000 T cells per well. (**b**) Estimated frequencies of autoreactive CD4^+^ T cells. Background (no Ag) was subtracted. (**a**,**b**) VIM n = 8 for LN, SLE and HCs, other antigens n = 6 for LN and n = 5 for SLE, and HCs, for TTR n = 5 for LN, SLE and HCs. (**c**) Correlation of calculated autoreactive T cell frequencies for VIM, ANXA2 and TTR (control Ag) with disease activity (SLEDAI, VIM n = 10, ANXA2 n = 8 TTR n = 8). The Mann–Whitney test was used for statistical analysis in (**b**), Spearman´s rank correlation in C (*p < 0.05), **p < 0.01, r = Spearman´s rank correlation coefficient). Horizontal lines depict the median. *No Ag* no antigen, *Ag* antigen, *VIM* Vimentin, *ANXA1* Annexin A1, *ANXA2* Annexin A2, *RPLP1* Ribosomal Protein P1, *RPLP2* Ribosomal Protein P2, *TTR* Transthyretin.

CD4^+^ T cell libraries were used to dissect the responses detected with flow cytometry and to evaluate the specific T cell reactivity to single antigens (for VIM all n = 8, other antigens n = 6 LN, n = 5 SLE and HCs, TTR all n = 5). Proliferation upon antigen challenge for all patients and antigens is shown in Fig. 3a.

Significant differences in autoreactive T cell frequencies between active LN and inactive SLE patients were found for VIM and ANXA2 (Mann–Whitney test, p = 0.03 and p = 0.02, respectively, Fig. 3b). Numerical differences, but no statistical significance, were observed when patients were compared to HCs (Mann–Whitney test, p = 0.08 and p = 0.1, respectively).

Reactive T cell frequencies for the control Ag TTR were comparable among all three groups. The frequencies of VIM and ANXA2 reactive T cells correlated with disease activity (SLEDAI) as determined by Spearman’s rank correlation coefficients (for VIM r = 0.57, p = 0.03; for ANXA2 r = 0.85, p = 0.002). In contrast, no correlation was found for TTR (r = − 0.36, p = 0.31; Fig. 3c).

### ***Detection of urinary CD4***^+^***T cells reactive to Vimentin in active LN patients***

CD4^+^ T cells are enriched in the urine of patients with active LN and have been shown to provide a better biomarker than proteinuria or sediment^21–23^. These cells most likely originate from the kidneys, since they express CXCR3 and seem to have mainly an effector memory phenotype^21,22^.

**Figure 4:**
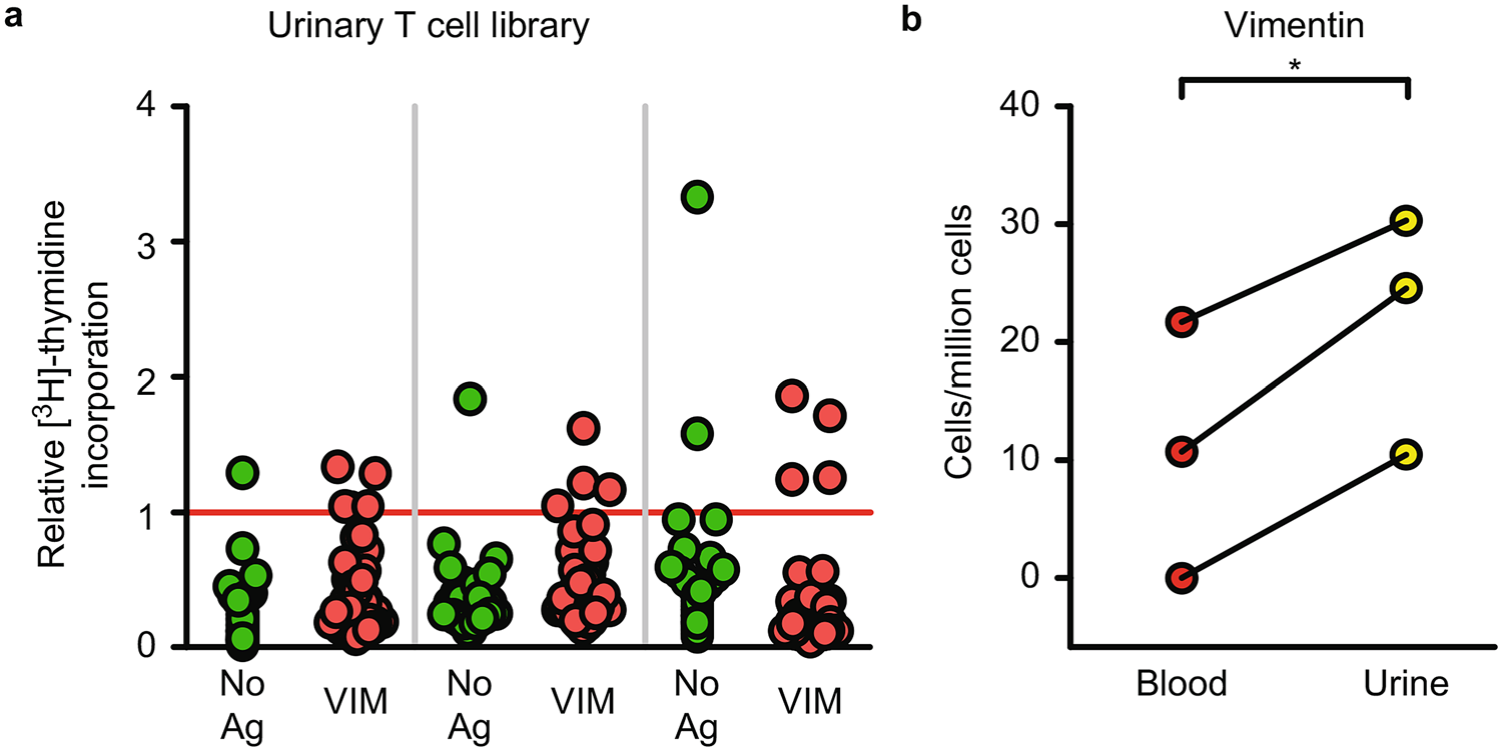
More Vimentin reactive CD4^+^ T cells were found in the urine of three patients with active LN. (**a**) Urinary CD4^+^ T cell libraries were stimulated with VIM or no Ag. Each column shows the relative [^3^H]-thymidine incorporation from a single patient, and each dot represents one microculture in a 96-well plate. Microcultures with a relative [^3^H]-thymidine incorporation above one (red line) were assumed to contain one reactive T cell. Libraries were set up with 2000 T cells per well. (**b**) Comparison of calculated frequencies of VIM-reactive CD4^+^ T cells in the blood and urine of LN patients (n = 3). Background (no Ag) was subtracted from each respective antigen group to calculate frequencies. Paired t test was used for statistical analysis. *No Ag* no antigen, *VIM* Vimentin.

We additionally succeeded in establishing urinary CD4^+^ T cell libraries from three patients with active LN. Libraries were stimulated with Vimentin, no antigens or SEB. A generally lower SEB proliferation index in urinary T cell libraries compared to blood was observed, which prompted us to accept a proliferation index of 3 as a criterion for exclusion of “unfit” microcultures (Supplemental Fig. 1f). Proliferation upon antigen challenge for all microcultures is shown in Fig. 4a. Significantly more Vimentin reactive T cells were found in urine than in blood of three LN patients (p = 0.02, paired t test), indicating kidney infiltration at the antigen-specific level (Fig. 4b). These results remained significant when applying the same microculture exclusion criteria as with blood cells (p = 0.02, paired t test, data not shown).

## Discussion

The concept of an antigen-specific CD4^+^ T cell response against kidney tissues in LN is favoured by reports of local MHC II upregulation, infiltration of oligoclonal CD4^+^ T cells in the interstitium of kidneys and a restricted renal T cell receptor repertoire^8,13,24^. Surprisingly, when we stimulated CD4^+^ T cells with kidney lysates from healthy individuals, no clear anti-renal T cell response was detectable with conventional flow cytometry. Additional testing of the most promising lysate in T cell libraries also provoked only marginal reactivity.

However, the lysates were manufactured from kidney tissues of patients who were not affected by SLE. In patients with active LN, the local inflammatory environment possibly provides different antigens. For B cells, this has already been shown in a study that described Vimentin as the major target of in situ humoral immunity in the tubulointerstitium of LN patients^18^. Assuming that B cell antigens also represent potential T cell antigens in SLE, we identified autoantibodies that have a strong association with LN development. We selected antibodies whose targets have increased expression in the kidney interstitium of LN patients, thus identifying potential renal antigens.

It needs to be stressed that the selected antigens are not exclusively expressed in the kidneys and therefore do not represent “pure” renal antigens. Additionally, potential further renal antigens may have been excluded in our antigen selection process. Therefore, the choice of antigens used is neither renal exclusive nor comprehensive. Nevertheless, the association of the respective autoantibodies with LN and the renal expression of these antigens, together with the presence of antigen-specific T cells, suggest that the respective autoreactive T cells may very well play a role in LN pathogenesis.

The detected CD4^+^ T cell response was mainly driven by IFN-γ-producing Th1 cells. This is in line with other publications that have also described Th1 cells as having an essential role in LN development^25,26^. Interestingly, a dominating Th1 response has been identified in humans both in blood and renal tissue^25^.

Furthermore, we detected IL-10-producing CD4^+^ T cells, which are likely to act as immunosuppressive agents^27^. However, due to the bipolar role of IL-10, these cells could also contribute to inflammation, possibly by activating B cells^28^. Enhanced autoantibody production by autoreactive B cells could then in turn lead to more severe renal inflammation and damage.

Determining the involvement of antigen-specific T cells in human disease pathogenesis in general is still an ongoing scientific challenge, and different approaches have been described in the literature. A recent paper analysed gluten-specific CD4^+^ T cells in patients with celiac disease via gluten tetramer binding and found overlapping clonotypes in gut and peripheral blood, which also persisted over time. During antigen challenge, the observed frequencies of CD4^+^ gluten-specific effector memory cells in blood ranged from approximately 10^1^ to 10^3^ cells per million CD4^+^ T cells, which is remarkably close to the frequencies we observed with kidney antigens in LN patients^29^.

Recently, the presence of CD4^+^ T cells reactive to citrullinated aggrecan epitopes in patients with rheumatoid arthritis (RA) has been described. In their study, the authors used tetramer staining to show that RA patients had significantly higher numbers of autoantigen-specific CD4^+^ T cells in peripheral blood than HCs^30^, again indicating a potential role for CD4^+^ T cells in autoimmune disease pathogenesis. Interestingly, frequencies ranged roughly from two to eight cells per million CD4 + T cells, which is on the same order of magnitude as our single-antigen T cell library results in LN patients.

With T cell libraries, we identified Vimentin and Annexin A2 as the main, but not exclusive, T cell targets. Given the potential contribution of a SLE associated hyperresponsiveness, cells were also stimulated with the control antigen TTR and no differences in reactive cell numbers were detected. Using the same experimental set-up, previous work from our group also found no differences between active and inactive SLE patients and healthy controls upon stimulation with the recall antigen MP65 from *candida albicans*^31^.

Annexin A2 also mediates the binding of beta 2-glycoprotein I to endothelial cells in antiphospholipid syndrome (APSN)^32^. In renal histopathological samples from LN and APSN patients, Annexin A2 has been described as being highly expressed in glomerular and peritubular capillaries and thus was associated with vascular lesions^33^. As mentioned before, epithelial cells in the kidneys have been described to express MCH II. Therefore, they might be responsible for the antigen presentation of Annexin A2 to autoreactive T cells in human LN. In contrast, Vimentin has been described to be mainly an interstitial antigen^18^. It could be speculated that Annexin A2 might particularly represent a vascular T cell target, while Vimentin might be more relevant in the interstitium. Potentially, this could contribute to the observation of intrarenal vascular changes typically associated with APSN in LN biopsies. However, further studies with clear histopathological focus are required to address this issue.

Interestingly, no major T cell response against ribosomal P proteins was detectable in our experiments. However, given the association of autoantibodies directed against these proteins with neuropsychiatric lupus, they might be more relevant in the pathogenesis of this SLE subtype^34^. An argument for this hypothesis could be that anti-ribosomal autoantibodies have been associated with a favourable renal function in LN if dsDNA antibodies were present^35^.

We additionally detected more Vimentin reactive T cells in urine than in blood of LN patients, indicating kidney infiltration at the antigen-specific level. Reports of oligoclonal T cell enrichment in LN kidneys determined by Vß TCR analysis and the detection of CXCR3-positive T cells in the urine of LN patients further support this concept^13,23^.

It is intriguing to assume that the local microenvironment in the kidneys of LN patients provides these renal antigens, thus driving infiltrating autoreactive CD4^+^ T cells to further boost the inflammatory process. They could then stimulate other immune cells on site, possibly via cytokine production.

Our study has several limitations. Restricted numbers of available PBMCs (peripheral blood mononuclear cells) per patient forced us to leave out a positive control in the ARTE experiments. Additionally, the number of SLE patients, especially those presenting with new renal involvement, had to remain limited. We used all CD3^-^ cells as antigen-presenting cells in T cell libraries, in contrast to the originally described procedure, which solely relied on monocytes^36^. Although T cell expanding conditions were used in CD4^+^ T cell libraries, especially in active SLE patients and in urinary T cell libraries, purity was not 100% when cultures were set up. Additionally, we only succeeded in establishing three urinary T cell libraries due to inherent urinary cell death. In contrast to the more or less dichotomous categorisation of T cells in being reactive/non-reactive, future investigations should address gradual stages of reactivity. To this end we are presently cloning different TCRs of potentially autoreactive T cells. Using this approach, we hope to further validate the current findings and better understand the autoantigen recognition process in the future. Finally, although our used controls make it unlikely that a SLE associated general hyperresponsiveness causes the observed results we cannot completely rule out a contribution of unspecific effects.

Nevertheless, we believe that despite these limitations, the consistent detection of renal autoantigen reactive T cells with two complementary methods strengthens our observations and the validity of our main findings.

Altogether, our results demonstrate that CD4^+^ T cells reacting with renally expressed antigens arise in the course of LN. These cells are mainly of the Th1 phenotype, very likely contribute to renal damage and may represent a promising treatment target.

## Methods

### Patients

Samples were collected after informed consent was obtained from patients with SLE at the Medical Department, Division of Rheumatology and Clinical Immunology and the Division of Nephrology and Internal Intensive Care at the Charité University Hospital. Ethics approval was obtained from the Ethics Commission of the Charité (number EAI/036/16). All experiments were performed in accordance with the relevant guidelines and regulations. This study was performed in accordance with the Declaration of Helsinki. Samples from healthy controls were obtained from voluntary donors after informed consent.

Samples from 57 SLE patients and 31 HCs were collected. Blood samples from 23 SLE patients and 12 HCs were used for stimulation with kidney lysates and analysis with flow cytometry. T cells from four SLE patients and five HCs were stimulated with kidney lysates in T cell libraries (Table 1). T cells from 16 active LN patients, 14 inactive SLE patients and 14 HCs were stimulated with kidney antigens and measured either with flow cytometry or T cell libraries (Table 2). For three patients, urinary T cells were also analysed in T cell libraries. The disease activity was determined with the systemic lupus erythematosus disease activity index (SLEDAI)^37^.

**Table 1:** Stimulation with kidney lysates.

	Flow cytometry		T cell library	
	SLE patients	Healthy controls	SLE patients	Healthy controls
Number of patients	23	12	4	5
Sex	20 f/3 m	9 f/3 m	1 f/3 m	2 f/3 m
Median age (range)	34 (22–70)	26 (22–31)	31 (26–62)	28 (23–35)
Disease activity (SLEDAI, median)	4 (0–26)	n.a	12 (8–14)	n.a

**Table 2:** Stimulation with kidney antigens (flow cytometry and T cell libraries).

	Active LN	Inactive SLE	Healthy controls
Number of patients	16	14	14
Sex	All female	All female	10 f/4m
Age (median)	32	30	26
Median disease activity (SLEDAI)	17	3	n.a
Treatment	6 × Cyclophosphamide 14 × Prednisone 4 × Azathioprine 6 × Hydroxychloroquine 1 × Rituximab 1 × Belimumab 1 × Mycophenolate-Mofetil	1 × Methotrexate 9 × Prednisone 7 × Azathioprine 10 × Hydroxychloroquine 1 × Rituximab 3 × Belimumab 1 × Mycophenolate-Mofetil	None

### Recruitment

All patients with SLE presenting in our hospital were eligible for this study. For some patients, this was the first presentation of systemic lupus erythematosus. Blood was obtained as soon as possible to minimize potential treatment effects on the experiments. For almost all active cases, this was before histopathological data confirmed the diagnosis. For most inactive patients, blood draws were performed during a routine visit in the outpatient clinic. These patients had been previously diagnosed with SLE. All patients fulfilled the 2019 European League Against Rheumatism/American College of Rheumatology Classification Criteria for Systemic Lupus Erythematosus^38^. Active renal involvement was defined by high overall disease activity (SLEDAI ≥ 10) and a current kidney biopsy (not older than 4 weeks) showing LN. In the absence of a current biopsy, active renal involvement was defined by high disease activity (SLEDAI ≥ 10) and at least two elements of the renal SLEDAI. Histopathological classification for all patients who were tested in the experiments with potential renal antigens can be found in Supplementary Table 1. Recent information on glomerular vs interstitial involvement is provided, if available.

### Stimulation with kidney lysates and measurement with flow cytometry

PBMCs were isolated from 10–40 ml of heparinized blood by Ficoll-Paque PLUS (GE Healthcare Europe, Freiburg, Germany) density gradient centrifugation. PBMCs were resuspended in RPMI 1640 medium with GlutaMAX containing 1% penicillin/streptomycin (both Life Technologies, Carlsbad, California, USA), 5% human AB serum (Sigma-Aldrich Chemie GmbH, Munich, Germany) and 1 µg/ml anti-human CD28 antibody (eBioscience, San Diego, California, USA) and distributed in a 96-well round-bottom plate (Greiner-Bio-One, Frickenhausen, Germany) in 100 µl medium at a concentration of 5 × 10^5^ PBMCs per well. Cells were then stimulated with three different kidney lysates at a concentration of 10 µg/ml. Lysates from normal kidney tissue (KID, PromoCell, Heidelberg, Germany), epithelial cells of the tubulus (EPI) and epithelial cells of the proximal tubulus (TUB, both ScienCell, Carlsbad, California, USA) were used. The lysates were dialysed prior to use with Slide-A-Lyzer MINI Dialysis Units and Slide-A-Lyzer MINI Dialysis Floats (both Thermo Scientific, Braunschweig, Germany). Cells were incubated for 6 h. For the last four hours, 2 µg/ml Brefeldin A (Sigma-Aldrich Chemie GmbH) was added. Cells were fixed with 2% paraformaldehyde. Saponin (0.5%) in PBS/BSA/AZID (Deutsches Rheuma Forschungszentrum, DRFZ, Berlin, Germany) for intracellular staining. The following anti-human antibodies were used: CD3-PE (clone BW264/56), CD69-FITC (clone FN50; both Miltenyi Biotec GmBH, Bergisch-Gladbach, Germany), CD3-PE (clone Ucht1), IFN-γ-Cy5 (clone 4SB3, all DRFZ), CD4-Pe-Cy7 (clone SK3), (BD Biosciences, San Jose, USA), CD40L-BV421 (clone 24–31), and IFN-γ-APC (clone 4SB3, both BioLegend Inc., San Diego, USA). Additionally, cells were stained with propidium iodide.

### Cells counting

Cells were counted either with a Casy cell counter (Schärfe System GmbH, Reutlingen, Germany) or a Guava easyCyte flow cytometer (Merck Millipore, Massachusetts, USA).

### Antigen identification

PubMed was searched for “lupus nephritis autoantibody” on the 8th of June 2015, including papers from 2011 to 2016. A review published in 2015 was used for autoantibodies identified prior to 2011^39^. In total, 430 papers were found. Evaluation of the abstracts found 33 papers that described potential human kidney antigens. These were chosen and read in detail^40–72^. Ten articles dealt with autoantibodies that were not already described in the review^63–72^. In total, 34 autoantibodies were found; 22 autoantibodies were selected for which specific molecular targets were reported, and 12 “autoantibody-hits” were discarded because the targets were not defined on a molecular basis (e.g., endothelial cells). The targets of the remaining 22 autoantibodies (82 genes) were analysed, the gene expression in the tubulointerstitium of patients was compared between patients with LN and HCs. Gene expression data were retrieved from the Geo database^73^. Twenty-one genes were found to have higher expression in the interstitium of patients with LN (p < 0.01; Mann–Whitney test). Nucleus-, blood-, and immune-associated genes were discarded as well as fibronectin, alpha actinin and laminin B1. Five target antigens remained: Vimentin, Ribosomal Protein P1, Ribosomal Protein P2, Annexin A1 and Annexin A2. Transthyretin was chosen as a negative control. With plasma concentrations of 20–40 mg/dl, it is a highly abundant protein in human blood and not differentially expressed in the kidneys of LN patients^73,74^.

### Antigen reactive T cell enrichment (ARTE)

Low frequencies and high background have hampered the identification and characterization of antigen-specific autoreactive Tcons. Recently, the antigen reactive T cell enrichment (ARTE) method has been developed to overcome these difficulties. We adapted the assay from the originally described procedure^19^. PBMCs were isolated from 40–50 ml of heparinized blood by Ficoll-Paque PLUS (GE Healthcare Europe) density gradient centrifugation. Cells were rested overnight in PBS/BSA (DRFZ). PBMCs were distributed on a 12-well plate (Greiner-Bio-One) in 1 ml of RPMI 1640 medium per well with GlutaMAX containing 1% penicillin/streptomycin (both Life Technologies), 5% human AB serum (Sigma-Aldrich Chemie GmbH), 1 µg/ml anti-CD28 (eBioscience) and 1 µg/ml anti-CD40 antibody (Miltenyi Biotec GmbH). Either no antigen or an antigen pool (Ag–P) containing Vimentin, Annexin A1, Annexin A2, Ribosomal Protein P1 and Ribosomal Protein P2 each at a concentration of 0.5 µg/ml was added. Because of limitations in available patient samples and the expectation of very few antigen-specific T cells, whole PBMCs were used for Ag–P stimulation only, and no further positive control was included. Cells were incubated for 7 h. A total of 2 µg/ml Brefeldin A (Sigma-Aldrich Chemie GmbH) was added after 5 h of incubation. Cells were harvested, counted with a Guava easyCyte Flow Cytometer (Merck Millipore, Massachusetts, USA) and enriched via CD154 MicroBeads and MS columns (both Miltenyi Biotec GmbH). The enriched fraction was fixed with 2% paraformaldehyde (DRFZ). Intracellular staining was performed with BD FACS Permeabilizing Solution 2 (BD Biosciences). The following anti-human antibodies were used: CD3-VioGreen (clone BW264/56), CD4-APC-Vio770 (clone M-T321), CD69-VioBlue (clone FN50), Biotin-PE, CD154-PE (clone 24–31; all Miltenyi Biotec GmbH), CD69-PerCP (clone FN50), IFN-γ-APC (clone 4S.B3), IL-10-PECy7 (clone JES3-9D7; all BioLegend Inc), IL-4-FITC (clone BS4), IL-17-PerCPCy5.5 (clone eBio64DEC17; both Life Technologies Europe BV), and IL-17-BV786 (clone N49-653, BD Biosciences).

### Calculation of cell frequencies

Cell frequencies in original samples were calculated by dividing the cell count of enriched autoreactive T cells by the CD3^+^CD4^+^ cell count in the original sample prior to enrichment, since the enrichment process analysing percentages in flow cytometry makes no sense (one needs to consider whole cell counts in enriched samples, not percentages). Background (no Ag) was then subtracted, whereas negative results were counted as zero. Then, log (x + 1) transformation was performed.

### T cell libraries

Again, the assay was adopted from the originally described procedure^36^. PBMCs were isolated from 40–50 ml of heparinized blood by Ficoll-Paque PLUS (GE Healthcare Europe) density gradient centrifugation. T cells were enriched via CD4 MicroBeads and LS columns. The negative fraction was then depleted via CD3 MicroBeads and LD columns (all Miltenyi Biotec GmbH). Purity was checked afterwards with CD3/CD4 staining and flow cytometry (median purity: LN 0.65; SLE 0.94; HCs 0.97). Because of limited access to patient PBMCs, the whole CD3- fraction was cryopreserved in liquid nitrogen and utilized as antigen-presenting cells. Cells were counted with a Casy cell counter (Schärfe System GmbH). A total of 2,000 CD4^+^ T cells were cultured per well in a 96-well round-bottom plate (Greiner-Bio-One) in 20 ml IMDM with GlutaMAX containing 1% penicillin/streptomycin (both Life Technologies), 2 µg/ml ciprofloxacin, 5% human AB serum, 1 µg/ml PHA, 1% MEM nonessential amino acid solution (all Sigma-Aldrich Chemie GmbH) and 1 × 10^5^ irradiated feeder cells/ml (self-prepared at the DRFZ) and 600 IU/mL IL-2 (Proleukin/Aldesleukin, Novartis Pharma GmbH, Nuremberg, Germany). The medium was exchanged after three and seven days. On day nine, the cultures were split into eight 96-well plates and stimulated once more with PHA and IL-2 for further expansion. After three days, the medium was exchanged, and the cells rested for at least four days. Then, new medium containing the CD3^-^ fraction and either no antigen, Vimentin (Orgentec Diagnostica GmbH, Mainz, Germany), Annexin A1, Annexin A2, Ribosomal Protein P1, Ribosomal Protein P2, or Transthyretin (Antibodies Online, Aachen, Germany) at a concentration of 0.5 µg/ml or staphylococcal enterotoxin B (SEB, Sigma-Aldrich Chemie GmbH) at a concentration of 1 μg/mL was added. Antigens were desalted with 0.5 ml Zeba Spin Desalting Columns (Thermo Fisher Scientific, Massachusetts, USA) prior to use. T cells from four SLE patients and five HCs were stimulated with lysates from normal kidney tissue (PromoCell) at a concentration of 10 µg/ml. Two days later, [^3^H]-thymidine (GE Healthcare UK Ltd., Little Chalfont, United Kingdom) was added, and proliferation was measured 16 h later in a MicroBeta^2^ Plate Counter (PerkinElmer Inc., Waltham, MA, USA). For three patients with active lupus nephritis, urinary T cells were enriched via CD4 MicroBeads (Miltenyi Biotec GmbH) in addition to blood T cells, expanded in libraries and stimulated with no antigen or Vimentin.

Microcultures with an SEB stimulation index below five were excluded from further statistical analysis, with the exception of urine-derived libraries, for which a stimulation index of three was used due to lower overall proliferation (Supplemental Fig. 1f). [^3^H]-Thymidine incorporation values were not normally distributed; therefore, a new analysis method was used (Supplemental Fig. 1e). Instead of comparing mean cpm values, a threshold was defined by calculating the median of the unstimulated samples and adding 5 × (3rd quartile–1st quartile). All data sets of each individual patient were then divided by the threshold, thus enabling data presentation and comparison with a relative [^3^H]-thymidine incorporation. Microcultures with a relative [^3^H]-thymidine incorporation above one were assumed to contain one reactive T cell. Autoreactive T cell frequencies were then calculated, and background (no antigen) was subtracted.

### Flow cytometry

Cells were measured using a BD FACSCanto II or BD LSRFortessa (BD Biosciences, San Jose, USA)) flow cytometer using FACSDiva software (BD Biosciences) or using a MACSQuant flow cytometer (Miltenyi Biotec GmBH) at the Flow Cytometry Core Facility (FCCF) of the German Rheumatism Research Center (DRFZ) in Berlin, Germany. Data were analysed with FlowJo Analysis software (Three Star, Ashland, USA).

### Statistical analysis

Statistical analysis was performed with GraphPad Prism version 5 for Windows (GraphPad Software., San Diego, California, USA, www.graphpad.com). The Mann–Whitney test was used to compare nonpaired samples. The Wilcoxon signed rank test was used to compare samples of single donors in different conditions. For the comparison of Vimentin-specific T cell frequencies in blood and urine, paired t tests were used. Nonparametric correlation was analysed using Spearman’s rank correlation coefficients.

## Supplementary information

Supplementary Information 1.

Supplementary Information 2.

## Data Availability

The datasets generated and analysed during the current study are available from the corresponding author on request.
