# Supplementary material for: Identification and characterization of antigen-specific CD4+ T cells targeting renally expressed antigens in human lupus nephritis with two independent methods
Source: Sci Rep. 2020 Dec 4;10:21312. doi: 10.1038/s41598-020-78223-3 (PMC7718878; doi:10.1038/s41598-020-78223-3)
Supplement: Supplementary file 1 — Supplementary Information 1. [file 41598_2020_78223_MOESM1_ESM.doc]

**Title:** Identification and characterization of antigen-specific CD4^+^ T cells targeting renally expressed antigens in human lupus nephritis with two independent methods

**Authors:** Sebastian Tesch, Dimas Abdirama, Anna-Sophie Grießbach, Hannah Brand, Nina Goerlich, Jens Y Humrich, Petra Bacher, Falk Hiepe, Gabriela Riemekasten, Philipp Enghard

**Supplemental Fig. 1.**

a. Flowchart depicting the search algorithm we used to identify renal target antigens. b. Comparison of tubulointerstitial gene expression between LN patients and HCs for the five identified antigens as well as the control antigen TTR. Data were retrieved from the Geo database (n = 15 HCs, n = 32 LN) c. and d. Flowcharts for antigen-specific T cell enrichment (C.) and T cell libraries (D.) e. Raw cpm-value data varied considerably (n = 24) between all patients f. The ratio of proliferation in response to SEB to no Ag was lower in urinary T cell libraries. Therefore, a lower exclusion criterion was applied in the statistical analysis (n = 3). g. Frequency of CD154^+^CD69^+^IFN-γ^+^ Tcons in SLE patients in response to stimulation with SEB. Cells were measured with conventional flow cytometry (n = 23). h. Calculated cell frequencies for CD4^+^ T cell libraries and stimulation with KID (n = 5 HCs, n = 4 SLE). i. Tcon frequencies after antigen-specific T cell enrichment for no Ag- and Ag-P-stimulated samples (n = 8 LN, n = 6 SLE and HCs). LN, lupus nephritis; HC, healthy control; TTR, transthyretin; cpm, counts per minute; SEB, staphylococcal enterotoxin B; no AG, no antigen; Tcon, conventional CD3- and CD4-positive T cells; KID, lysates from normal kidney tissue; Ag-P, antigen pool.

**Supplementary Table 1.** Patient characteristics for stimulation experiments with potential renal antigens

| **Group** | **SLEDAI** | **Age** | **Sex** | **Daily prednisone dose** | **LN class if applicable** | **Glomerular involvement** | **Interstitial involvement** | |
| --- | --- | --- | --- | --- | --- | --- | --- | --- |
| LN | 20 | 29 | f | 0 | IV/V | inflamed | interstitial inflammation, no tubulitis |  |
| LN | 20 | 19 | f | 50 | IV | inflamed | no inflammation |  |
| LN | 20 | 20 | f | 50 | IV | inflamed | interstitial inflammation and tubulitis |  |
| LN | 18 | 25 | f | 20 | IV | n.a. | n.a. |  |
| LN | 20 | 29 | f | 250 | IV | inflamed | no inflammation |  |
| LN | 20 | 36 | f | 60 | IV | inflamed | interstitial inflammation, no tubulitis |  |
| LN | 14 | 18 | f | 250 | IV | inflamed | interstitial inflammation and tubulitis |  |
| LN | 10 | 54 | f | 8 | (III/V) |  |  |  |
| LN | 16 | 56 | f | 5 | (IV) |  |  |  |
| LN | 10 | 52 | f | 125 | n.a |  |  |  |
| LN | 12 | 34 | f | 13 | (IV) |  |  |  |
| LN | 14 | 64 | f | 10 | IV | inflamed | interstitial inflammation, no tubulitis |  |
| LN | 18 | 40 | f | 10 | IV | inflamed | no inflammation |  |
| LN | 12 | 56 | f | 10 | (IV/V) |  |  |  |
| LN | 12 | 19 | f | 20 | (IV) |  |  |  |
| LN | 12 | 24 | f | 10 | (IV) |  |  |  |
| SLE | 2 | 31 | f | n.a. | n.a. |  |  |  |
| SLE | 4 | 38 | f | 5 | n.a. |  |  |  |
| SLE | 1 | 26 | f | n.a. | (V) |  |  |  |
| SLE | 4 | 22 | f | 8 | (II/V) |  |  |  |
| SLE | 2 | 29 | f | 20 | (IV) |  |  |  |
| SLE | 2 | 29 | f | n.a. | (IV) |  |  |  |
| SLE | 4 | 23 | f | 10 | n.a. |  |  |  |
| SLE | 4 | 29 | f | 4 | n.a. |  |  |  |
| SLE | 2 | 37 | f | 5 | n.a. |  |  |  |
| SLE | 4 | 42 | f | 10 | n.a. |  |  |  |
| SLE | 4 | 38 | f | 5 | (IV/V) |  |  |  |
| SLE | 6 | 19 | f | n.a. | n.a. |  |  |  |
| SLE | 2 | 40 | f | n.a. | n.a. |  |  |  |
| SLE | 2 | 35 | f | 5 | (IV) |  |  |  |

Results for biopsies older than four weeks are shown in brackets. For one active patient*,* no information on glomerular vs. interstitial involvement was available due to outward biopsy analysis.
